# IL-20 Subfamily Biological Effects: Mechanistic Insights and Therapeutic Perspectives in Cancer

**DOI:** 10.3390/ijms26157320

**Published:** 2025-07-29

**Authors:** Valentina Maggisano, Maria D’Amico, Saveria Aquila, Francesca Giordano, Anna Martina Battaglia, Adele Chimento, Flavia Biamonte, Diego Russo, Vincenzo Pezzi, Stefania Bulotta, Francesca De Amicis

**Affiliations:** 1 Department of Health Sciences, University “Magna Graecia” of Catanzaro, 88100 Catanzaro, Italy; 2 Department of Pharmacy and Health and Nutritional Sciences, University of Calabria, 87036 Rende, Italy; 3 Centro Sanitario, Università della Calabria, 87036 Rende, Italy; 4 Department of Experimental and Clinical Medicine, University “Magna Graecia” of Catanzaro, 88100 Catanzaro, Italy

**Keywords:** inflammation, cytokines, cancer progression

## Abstract

The interleukin-20 (IL-20) cytokine subfamily, a subset of the IL-10 superfamily, includes IL-19, IL-20, IL-22, IL-24, and IL-26. Recently, their involvement in cancer biology has gained attention, particularly due to their impact on the tumor microenvironment (TME). Notably, IL-20 subfamily cytokines can exert both pro-tumorigenic and anti-tumorigenic effects, depending on the context. For example, IL-22 promotes tumor growth by enhancing cancer cell proliferation and protecting against apoptosis, whereas IL-24 demonstrates anti-tumor activity by inducing cancer cell death and inhibiting metastasis. Additionally, these cytokines influence macrophage polarization—an essential factor in the immune landscape of tumors—thereby modulating the inflammatory environment and immune evasion strategies. Understanding the dual role of IL-20 subfamily cytokines within the TME and their interactions with cancer cell hallmarks presents a promising avenue for therapeutic development. Interleukin-20 receptor antagonists are being researched for their role in cancer therapy, since they potentially inhibit tumor growth and progression. This review explores the relationship between IL-20 cytokines and key cancer-related processes, including growth and proliferative advantages, angiogenesis, invasion, metastasis, and TME support. Further research is necessary to unravel the specific mechanisms underlying their contributions to tumor progression and to determine their potential for targeted therapeutic strategies.

## 1. Introduction

Over the past century, advancements in tumor biology knowledge have underscored the intricate relationship between the immune system and both healthy and malignant cells. The immune system acts to identify and eliminate transformed cells, and impaired immune responses can correlate with high cancer incidence. However, the constant interplay between tumor processes and the host immune response shapes cancer development and progression. In this context, the features of cancer cells, such as the evading immune destruction and fostering a pro-tumoral inflammatory environment, play a fundamental role [1,2].

One of the clearest indications that a disrupted immune response contributes to cancer development is the high incidence of cancer in tissues experiencing chronic inflammation [3]. The process of tumorigenesis in such tissues is driven by a persistent inflammatory response, leading to the accumulation and activation of various stromal cell types in theTME. These cells’ normal roles in maintaining homeostasis become maladaptive, creating a niche that supports tumor development.

The cellular and non-cellular stromal components of TME co-evolve with tumor cells. The dynamic and mutualistic interactions within the TME are most recently recognized as key determinants of cancer hallmarks [4].

The concept of cancer hallmarks refers to the essential characteristics that enable cancer cells to grow uncontrollably and spread, thus representing the acquired abilities of cells as they pass from normal to neoplastic growth, contributing to the development and progression of malignant tumors. Initially, six such hallmarks were identified [5]. The number of cancer hallmarks has expanded since their initial identification. They now include maintaining proliferative signaling, avoiding growth suppressors, resisting cell death, enabling limitless replication, inducing or accessing growth factors, triggering invasion and metastasis, disrupting cellular metabolism, evading immune destruction, promoting genome instability and mutation, and fostering tumor-promoting inflammation [6,7]. The TME contributes to the acquisition and maintenance of these hallmarks to varying degrees. Specifically, if inflammation is not properly resolved, the immune cell infiltration and cytokine secretion are maintained long-term. This leads to harmful chronic inflammation, usually of lower intensity and longer duration than the physiological inflammation in response to damage, that is found in cancer and other pathological conditions.

The major regulators of inflammation are cytokines, small secreted molecules involved in cell-to-cell communication, and chemokines, which mediate the migration and recruitment of leukocytes [8]. Cancer and stromal cells release a variety of signaling molecules, including ILs, a type of cytokine, which facilitate the infiltration and regulation of immune cells within the TME. The recruited leukocytes and other stromal cells subsequently produce a range of pro-inflammatory cytokines, creating positive feedback loops. These inflammatory responses activate transcription factors that are extensively involved in cancer development and progression, such as signal transducer and activator of transcription 3 (STAT3), hypoxia-inducible factor 1 alpha (HIF1α), and nuclear factor-κB (NF-kB). The activity of these transcription factors further stimulates the production of inflammatory mediators [9]. Consequently, chronic inflammation significantly contributes to the plasticity of the TME, creating a pro-tumorigenic milieu that promotes tumorigenesis, immunosuppression, and cancer progression [10]. Indeed, due to the biodiversity within cancer cell subpopulations and TME, the overall survival and response to therapeutics differ greatly among patients.

The challenge is targeting both the TME and the tumor; thus, strategies targeting molecular and cellular relationships that promote cancer progression hold considerable promise.

Interleukins, important players in a large cytokine network, are among key elements that orchestrate the TME and govern tumor–immune cell crosstalk [11]. Recently, the IL-20 subfamily of cytokines has garnered attention in cancer research due to its ability to influence host immune responses, modulate the activities of cells and molecules in the TME, and directly affect various premalignant and malignant tumors [12]. Notably, several members of the IL-20 subfamily are emerging as key players in the modulation of TME and cancer cell interactions [13].

The IL-20 family refers to a subgroup of ILs within the IL-10 cytokine family, based on structural, genetic, and functional similarities. From a structural perspective, the cytokines belonging to the IL-20 family all share a four α-helix bundle structure, which is typical of class II cytokines. In addition, they are heavily glycosylated and exhibit very similar tertiary structures, suggesting a close evolutionary and functional relationship among them [14].

Among them, IL-22, a prominent member of the IL-20 family, can be produced by several hematopoietic cell types and is a crucial effector cytokine produced by T helper (TH) 17 cells. These cells are increasingly recognized as a significant component of tumor-infiltrating lymphocytes [15], and their role in tumor progression varies widely depending on the type of immune cell. Uniquely, IL-22 acts exclusively on non-hematopoietic cells, distinguishing it from other immune-cell-derived cytokines [16]. Recent research on the role of IL-22 in tumor angiogenesis offers deeper insights into how TH17 cells contribute to cancer development and progression [17]. However, there are few reports on the direct effect of IL-22 on the immune cells in the TME, which may represent a direction of future research. Conversely, increased expression and secretion of IL-24, another member of the IL-20 subfamily, was evidenced in macrophages polarized into a pro-inflammatory phenotype that induces apoptosis in gastric cancer (GC) cells [18].

More recently, preclinical basis supports therapeutic approaches aimed at targeting the immunomodulatory effects of the specific IL-20 family, including several members whose expression and signaling are frequently dysregulated across various human cancer types. In preclinical models of tumorigenesis in the intestine, blocking IL-22 or its receptor suppresses tumor growth and enhances the response to immunotherapy [19]. Although preclinical models show promising results, the current clinical effectiveness of molecules that inhibit ILs in metastatic cancer remains modest [20]. Nonetheless, ongoing clinical trials are exploring the use of IL blockers in combination with existing anticancer drugs across various cancer types [21].

This review aims to highlight the latest findings on the role of IL-20 subfamily members within the TME, with a particular focus on their involvement in key cancer hallmarks, proliferation, resistance to cell death, angiogenesis, migration, invasion, and the development of metastases. We further summarized therapeutic perspectives under investigation involving IL-20 subfamily members in the field of cancer.

## 2. The IL-20 Subfamily Members, Receptors, and Signaling

**Figure 1:**
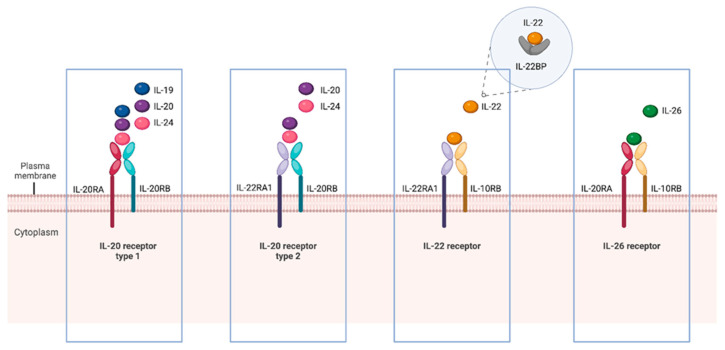
The IL-20 cytokine subfamily members and their corresponding receptors. This group includes IL-19, IL-20, IL-22, IL-24, and IL-26. These cytokines interact with receptors that consist of either the IL-20RB or IL-10RB, in combination with either the IL-20RA or IL-22RA1. Moreover, IL-22BP, which structurally resembles IL-22RA1, acts as a soluble receptor for IL-22. Scheme created using BioRender.com.

A subgroup of cytokines known as the IL-20 subfamily has been identified with complex and sometimes contradictory roles in different healthy and malignant cell functions, with effects on TME and cancer hallmarks. This subfamily comprises cytokines such as IL-19, IL-20, IL-22, IL-24, and IL-26, produced mainly by immune cells, T cells, B cells, Natural killer (NK) cells, innate lymphoid cells, monocytes, macrophages, and dendritic cells (DC), and is involved in the development of hematopoietic cells. IL-19, IL-20, and IL-24 are expressed particularly by TH type 2 cells, which orchestrate protective type 2 immune responses and protective function in some cancers [22] but also contribute to chronic inflammatory diseases. Primarily, cells of the lymphoid lineage, encompassing cells of both the innate and adaptive immune systems, including αβ T cells, γδ T cells, NKT cells, and innate lymphoid cells (ILCs), produce IL-22. However, some studies have also described macrophages, neutrophils, and fibroblasts [23] producing IL-22. IL-26 is expressed only in humans, and its main cellular sources include human T cells, while it is poorly expressed in B cells or monocyte lines and very few carcinoma lines examined [24]. IL-22 and IL-26 can also be expressed by keratinocytes [25] and airway epithelial cells [26] and act on endothelial cells [27], suggesting the possible reciprocal interaction between immune cells and tissue cells through the production of these cytokines. Given the pattern of their production, each IL-20 subfamily member interacts with distinct expressed heterodimeric receptors, namely IL-20 receptor type-1 (IL-20R1), IL-20 receptor type-2 (IL-20R2), IL-22 receptor (IL-22R), and IL-26 receptor (IL-26R) to exert specific biological effects (Figure 1). These specific receptors consist of combinations of shared subunits, including IL-20 receptor α-subunit (IL-20RA), IL-20 receptor β-subunit (IL-20RB), IL-10 receptor β-subunit (IL-10RB), and IL-20 receptor α1-subunit (IL-22RA1) [12]. Specifically, IL-19, IL-20, and IL-24 utilize the IL-20RA/IL-20RB receptor complex, while IL-20 and IL-24 can also engage the IL-20RB/IL-22RA1 complex. IL-22 and IL-26, on the other hand, signal through the IL-22RA1/IL-10RB and IL-20RA/IL-10RB receptor complexes, respectively. Additionally, IL-22 can interact with a soluble binding protein known as IL-22 binding protein (IL-22BP or IL-22RA2), which modulates IL-22 signaling by sequestering it. IL-22BP has a higher affinity for IL-22 than the membrane-bound IL-22RA1/IL-10RB receptor complex, resulting in the effective neutralization of IL-22 and inhibition of its downstream signaling [28].

The receptor subunits for IL-20 subfamily cytokines are primarily expressed on epithelial and endothelial cells, with limited expression on immune cells under steady-state conditions [29]. However, emerging evidence suggests that under pathological conditions, certain immune cells (e.g., monocytes, macrophages, or activated T cells) may upregulate these receptors, allowing for context-dependent, direct cytokine signaling [30]. The distinctive expression patterns of these receptors contribute to the unique biology of IL-20 subfamily cytokines. For example, IL-20RA and IL-22RA1 expression is confined to specific tissues and is essentially absent from hematopoietic lineages; thus, the selective distribution underscores the specialized roles of these cytokines in tissue homeostasis [23] and in several diseases, including cancer. For instance, there is notable upregulation of IL-20RA expression in pancreatic cancer (PC) [31], breast cancer (BC) [13], and non-small cell lung cancer (NSCLC), suggesting its potential role in cancer development and progression [32]. A recent study identified that IL-20RA is significantly expressed in colorectal cancer (CRC) tumor tissues and is associated with advanced stages of the disease [30]. These IL-20 family receptors play a tumor-intrinsic role and impact the ability of various immune cells to mount an effective anti-tumor response. In many cases, the immunomodulatory effects of IL-20 receptors in cancer are mediated indirectly: by acting on tumor or stromal cells to modulate the secretion of secondary mediators, alter antigen presentation, or reshape the TME in ways that influence immune cell recruitment, activation, or suppression [32].

While cytokines of the IL-20 subfamily can bind to individual receptor subunits, their interaction with the heterodimeric complex is essential for proper signal transduction [33]. Upon binding to their respective receptor complexes, all IL-20 subfamily members initiate activation of the Janus kinase (JAK) and STAT pathway, with a particular emphasis on STAT3, aberrantly hyperactivated in many types of cancer and generally associated with a poor clinical prognosis [34,35]. In the TME, inflammatory signals may upregulate IL-20R components on monocytes, macrophages, and DCs, enhancing their responsiveness to IL-20. This signaling, largely mediated through the JAK/STAT3 pathway overactivation, likely results in the suppression of antitumor immunity [36]. Simultaneously, STAT3 enhances the modulation of regulatory T (Treg) cells and myeloid-derived suppressor cells (MDSCs) [37]. Together, these actions contribute to a strongly immunosuppressive TME.

IL-20 subfamily cytokines are produced by various immune cells, and these same immune cells typically lack the receptor components necessary to respond to the cytokines they secrete. In contrast, IL-20 receptor complexes are primarily expressed on non-hematopoietic cells, such as cancer cells, fibroblasts, and epithelial cells within the tumor stroma. As a result, IL-20 cytokines act primarily on these target cells to induce gene expression programs that indirectly suppress antitumor immunity. For example, IL-20 signaling in tumor or stromal cells can drive the expression of Vascular Endothelial Growth Factor (VEGF), Transforming Growth Factor Beta (TGF-β), and Programmed Death-Ligand 1 (PD-L1), which promote immune evasion, angiogenesis, and a tolerogenic microenvironment. Thus, while the cytokines activate a shared JAK/STAT pathway, their downstream effects vary depending on cell type and receptor availability.

**Figure 2:**
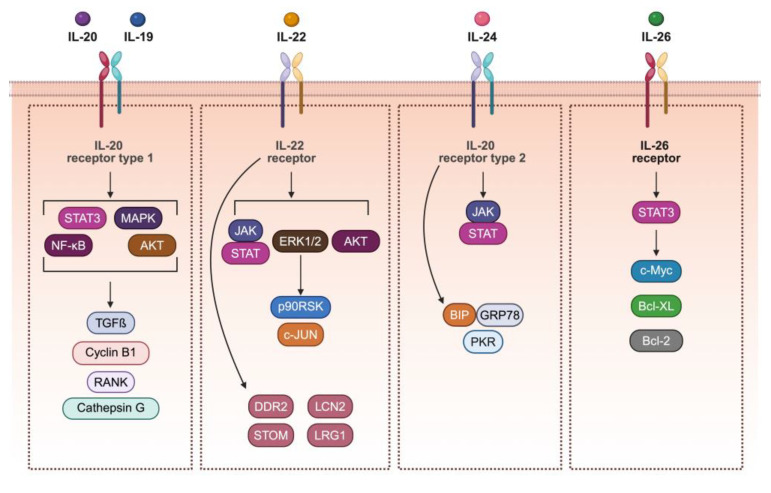
The IL-20 cytokine subfamily and the induced signaling in several cancer cell models. Scheme created using BioRender.com.

Specifically, IL-19 and IL-20 induced phosphorylation of STAT3, p38 mitogen-activated protein kinase (MAPK), JUN N-terminal kinase (JNK), Extracellular signal-Regulated Kinases 1 and 2 (Erk1/2), Akt, and Nuclear Factor kappa B (NF-κB) (Figure 2) in different human cancers, such as esophageal [38] and prostate [39] cell lines. In a rat hepatoma cell line, IL-22 was found to activate JAK1 but not JAK2, and STAT [40] and the three major MAPK pathways [40]. Notably, IL-22 signaling initiates a negative feedback loop by promoting the degradation of IL-22RA1 via the proteasome pathway [41]. Recent findings suggest that in a CRC cell model with LS174T cells, IL-22, through Erk1/2-mediated downstream regulation of its target, the p90 ribosomal S6 kinase (p90RSK), activates c-Jun and transcriptional changes in Tripartite motif containing 15 (TRIM15) and Stomatin (STOM), well-known for its modulatory actions of acid-sensitive ion channels. Additionally, IL-22 upregulates expression of Erk1/2-independent genes such as *Discoidin domain receptor tyrosine kinase 2 (DDR2)*, *Lipocalin 2 (LCN2)*, and *Leucine-rich alpha-2-glycoprotein 1* (*LRG1*), known for their involvement in the modulation of specific cancer hallmarks [42].

In cancer cells, IL-24 exerts its biological effects also via non-canonical JAK/STAT pathway activation through interaction with other intracellular molecular partners, including endoplasmic reticulum (ER)–resident chaperone BiP/GRP78 and dsRNA-activated protein kinase (PKR) [43].

IL-20R2 and IL-10R1, which are two essential receptors for IL-26 signaling, were expressed in GC cells. IL-26 activated STAT1 and STAT3 signaling; however, the upregulation of the expression of Bcl-2, Bcl-xl, and c-myc indicated that the effect of IL-26 is mediated by STAT3 activation [44].

## 3. Expression of IL-20 Subfamily Members and Their Receptors in Association with Specific Cancers

The role of IL-20 subfamily cytokines in cancer is complex and still under investigation, with potential variations depending on specific cancer types. While some studies suggest that these cytokines may affect tumor progression, influencing growth, invasion, and metastasis, the exact mechanisms remain unclear and subject to debate.

Various research efforts, including molecular profiling of specific cancer types, have provided insights into the potential correlation between IL-20 subfamily members’ expression profiles and disease outcomes, such as disease-free survival and clinicopathological factors [45].

### 3.1. IL-19

IL-19 has emerged as a key player in TME modulation and cancer progression. IL-19 is primarily involved in promoting an anti-inflammatory environment, which may facilitate tumor growth and metastasis. Elevated levels of IL-19 have been associated with various cancers, including BC and glioblastoma multiforme (GBM). Moreover, IL-19 contributes to an immunosuppressive tumor microenvironment by promoting the polarization of tumor-associated macrophages (TAMs) toward the M2 phenotype, which supports tumor growth and suppresses anti-tumor immune responses [46]. Of note, this effect is likely indirect, as macrophages do not express IL-20R components at functionally relevant levels.

A recent study by Small et al. revealed that the *IL-19* cytokine gene is activated in both mouse and human cells following various types of cellular stress, including DNA damage [47]. The study found that *IL19* expression is regulated by cyclic GMP-AMP synthase–stimulator of interferon genes pathway (cGAS-STING) or the kinase JNK, depending on the type of damaging stimulus. These findings indicate that IL-19 plays a role in initiating proinflammatory cytokine production and suggest that targeting IL-19 could be a potential therapeutic strategy for managing DNA damage responses in cancer patients [47]. In this regard, several studies demonstrate increased *IL19* expression in cancer specimens. A study utilizing tissue microarray technology and immunohistochemical analysis with an anti-IL-19 monoclonal antibody examined the expression of *IL-19* in both healthy and neoplastic tissues. The results indicated that IL-19 protein was present in healthy tissues. Additionally, positive IL-19 staining was observed in various tumor cells, notably in squamous cell carcinoma (SCC) of the skin, tongue, esophagus, and lung. This staining is associated with the activation of intracellular signaling pathways in cell lines derived from SCC of oral cavity tumor tissues. In particular, esophageal cancer cells express both IL-19 and its receptor IL-20R1/IL-20R2, suggesting a potential autocrine signaling loop. These observations suggest possible biological functions and clinical association of IL-19 in SCC tumors [48].

Elevated serum IL-19 levels correlate with lymph node metastasis and advanced stages in BC, suggesting its potential as a circulating biomarker of disease progression. High expression of *IL-19* in tumor tissue has been found to correlate with tumor metastasis and clinical staging. IL-19 directly influences esophageal cancer progression, inducing the expression of inflammatory mediators. In vivo studies using an anti-IL-19 monoclonal antibody—which binds directly to IL-19 and prevents its interaction with the IL-20R1/IL-20R2 receptor complex—have demonstrated suppression of tumor growth in esophageal squamous cell carcinoma. This blockade led to reduced expression of IL-19-regulated genes such as *TGF-β*, *Matrix Metalloproteinase-1* (*MMP-1*), *CXCR4*, and *Cyclin B* (*CCNB1*), highlighting the therapeutic potential of IL-19 inhibition in esophageal cancer [38]. Similarly, in BC, high *IL-19* expression in tumor tissue is associated with poor clinical outcomes. IL-19 emerges as a key mediator in BC pathogenesis, with its expression correlating with lymph node metastasis and distant metastasis. Patients with low *IL-19* expression exhibit higher disease-specific survival and metastasis-free survival compared to those with high *IL-19* expression. Thus, targeting IL-19 holds promise as a therapeutic strategy in BC treatment [49].

### 3.2. IL-20

The proinflammatory cytokine IL-20 has gained attention for its role in cancer biology [50]. IL-20, through IL-20R, influences various immune and non-immune cells and contributes to a pro-tumorigenic microenvironment. This occurs indirectly by inducing the expression of chemokines and cytokines that promote the recruitment and accumulation of immunosuppressive cells, including MDSCs and TAMs [13].

Elevated IL-20 levels have been observed in several malignancies. Expression of *IL-20* and all its receptor chains is increased in breast tumor tissue and correlates with a poorer prognosis [51]. Additionally, IL-20 is associated with muscle-invasive bladder cancer [52], while hepatocellular carcinoma (HCC) tumor tissue expressed higher levels of IL-20 than did non-tumor tissue. High *IL-20* expression in HCC was correlated with poor overall survival [53]. Elevated IL-20 levels in tumor tissue correlate with poor overall survival in patients with pancreatic ductal adenocarcinoma (PDAC).

Research has demonstrated that IL-20 and its receptor complex are frequently dysregulated in NSCLC through epigenetic mechanisms. In NSCLC tumor samples and cell lines, *IL-20RB* and *IL-22R1* are often overexpressed, while *IL-20RA* expression is suppressed, partly due to DNA CpG methylation and histone modifications. The epigenetic silencing of *IL-20RA* has been correlated with poorer disease-free survival in NSCLC, suggesting that loss of IL-20 signaling may contribute to tumor progression. Intriguingly, *IL-20RA* is normally expressed in immortalized human bronchiolar epithelial cells, suggesting a potential role in lung tissue homeostasis [32].

### 3.3. IL-22

There is controversial information regarding the prognostic significance of IL-22 in cancer [54]. IL-22 plays a dual role in tumorigenesis: while short-term IL-22 production helps protect against genotoxic stress, prolonged or uncontrolled IL-22 activity can facilitate tumor progression. Consequently, precise regulation of IL-22 is crucial. Particularly, IL-22-producing cells are associated with various human diseases, including several malignancies, such as lung, liver, gastric, colon, and pancreatic cancers, showing elevated IL-22 levels. In lung cancer (LC), studies have revealed increased IL-22 levels in patient sera and infiltration of IL-22-positive cells into primary tumors [55]. Additionally, higher *IL-22R1* mRNA expression is associated with worse recurrence-free survival in LC, indicating the importance of IL-22 in Kras-mediated lung tumorigenesis. Targeting IL-22 may offer a promising therapeutic approach for Kras-mutant LC, given its diverse biological effects on tumor cell phenotype [56].

Recent reports indicate that the presence of IL-22-producing T cells correlates with positive clinical outcomes in human CRC. The prognostic significance of *IL-22* expression was examined in large patient cohorts using two tissue microarrays to explore how IL-22 might affect the composition of the TME [57]. Further evidence demonstrates the role that IL-22 may play in inflammation-driven tumorigenesis and provides new insights into the aberrant cytokine signaling in oncogenic K-ras-associated colorectal carcinogenesis. Authors report that tissue IL-22 levels were significantly raised in the K-ras negative group compared to the CRC patients bearing the K-ras mutation [58]. Murine models suggest that uncontrolled IL-22 production promotes CRC development [19], potentially by directly affecting stem cells [59].

Gastric tumors exhibit increased infiltration of IL-22+ CD4+ T cells, with infiltration levels correlating with tumor stage and predicting poor prognosis [60]. Patients with GC also display elevated circulating IL-22-producing T cells, which correlate positively with disease progression and inversely with survival. Notably, increased *IL-22RA1* gene copy number and mRNA expression have been observed in primary tumor tissues, suggesting a potential for heightened IL-22 responsiveness in tumor cells. While receptor overexpression is not always required for cytokine signaling, elevated IL-22RA1 levels may enhance the sensitivity or magnitude of IL-22–driven responses, possibly amplifying downstream pro-tumorigenic pathways in the TME [61].

In both HCC and multiple myeloma, IL-22 appears to play a potential role in disease progression. In HCC, IL-22 is significantly upregulated in tumor-infiltrating leukocytes compared to peripheral lymphocytes, particularly in patients with advanced-grade tumors. Similarly, in multiple myeloma, elevated IL-22 levels are observed during active disease and decline following remission [62]. IL-22 acts on bone marrow stromal cells (BMSCs), which express *IL-22RA1*, leading to enhanced secretion of pro-survival and growth-promoting factors, such as IL-6 and VEGF, that support myeloma cell proliferation and survival. Furthermore, IL-22 contributes to immune evasion by promoting an immunosuppressive niche that limits cytotoxic T cell and NK cell function. These findings suggest that IL-22 plays an indirect but critical role in shaping the TME to favor multiple myeloma progression, and may represent a potential therapeutic target or biomarker of disease activity.

A recent study utilizing mouse models for PDAC discovered that myeloid cells expressing the IL-22 receptor (IL-10R2/IL-22R1) are prevalent in the blood of PDAC patients. These IL-10R2+ myeloid cells were found to predict tumor recurrence earlier than the traditional cancer marker CA19-9 in patients who had undergone pancreatectomy [63]. Additionally, *IL-22RA1* is highly, though variably, expressed in PC cells, with elevated levels linked to poorer patient prognosis. PC cells with high *IL-22RA1* expression demonstrated greater stemness potential and tumorigenicity. Importantly, IL-22 was found to enhance PC stemness through IL-22RA1/STAT3 signaling, elucidating the role of microenvironmental factors in regulating cancer stemness [64].

### 3.4. IL-24

Despite being identified several decades ago as melanoma differentiation-associated gene-7 (MDA-7), IL-24 continues to reveal new aspects of its role in normal physiology and various human diseases, including cancer. Specifically, early investigations revealed a notable decline in both gene and protein expression levels of IL-24 within the invasive regions of human melanomas [65,66].

IL-24 protein expression was evident in normal melanocytes, skin smooth muscle cells, and early-stage melanomas, but was notably absent in more advanced tumors [67]. This decline in IL-24 expression appears to coincide with the progression of melanoma. Intriguingly, introducing the *IL-24* gene into melanoma cells resulted in a suppression of cell growth and colony formation, suggesting a potential therapeutic avenue.

Recent research has extended beyond melanoma, exploring *IL-24* expression in various tumor types to assess its prognostic value. In BC, diminished *IL-24* expression levels are associated with unfavorable clinical outcomes, including lymph node metastasis and reduced disease-free survival [68]. Similarly, in rectal cancer, *IL-24* expression shows an inverse correlation with the extent of lymph node involvement, suggesting a potential role as a prognostic marker in this malignancy as well [69]. Mechanistically, IL-24 enhances anti-tumor immunity by promoting the secretion of pro-inflammatory cytokines e.g., Interferon (IFN-γ), Tumor necrosis factor (TNF-α), activating immune effector cells such as CD8^+^ T cells and NK cells. IL-24 also modulates the TME by suppressing angiogenesis and reducing immunosuppressive factors, contributing to a more favorable immune landscape for tumor control [70].

Additionally, IL-24 receptors exhibit widespread expression, encompassing various cancer types such as melanoma, prostate, pancreatic, fibrosarcoma, ovarian, and BC. This broad expression profile indicates that IL-24 could play such a role in varied oncological processes [71].

### 3.5. IL-26

IL-26 was initially identified for its role in immune responses. Recently, IL-26 has gained attention for its involvement in cancer development, with elevated levels observed in various tumor tissues [72].

A study investigating *IL-26* expression in HCC revealed that elevated IL-26 levels are associated with poorer recurrence-free and overall survival in HCC patients following resection therapy. Patients with tumors larger than 5 cm, TNM stages III–IV, and microvascular invasion exhibited higher *IL-26* expression, underscoring the prognostic significance of this cytokine in predicting adverse outcomes in HCC patients [73]. In GC, *IL-26* mRNA and serum levels are significantly higher in patients compared to healthy controls, suggesting its involvement in GC pathogenesis and potential as a prognostic indicator and therapeutic target [74]. Additionally, an analysis of IL-26 serum levels in 302 GC patients across various stages (I, II, III, and IV) demonstrated that IL-26 levels were significantly higher in patients with malignant tumors compared to those with benign conditions. Furthermore, higher IL-26 levels were associated with more advanced clinical stages of the disease [75].

Triple-negative breast cancer (TNBC) cells are exposed to IL-26 secreted by infiltrating CD4+ T cells and macrophages in the TME. IL-26 was also distinctly detected in tumor-infiltrating lymphocytes (TILs) of not only TNBC but also HER2-positive and Luminal breast tumors. IL-26 influences TNBC progression and lung metastatic growth, with elevated transcripts found in TNBC specimens [76].

Furthermore, patients with malignant pleural effusion who had higher IL-26 levels exhibited shorter survival times compared to those with lower IL-26 concentrations. This finding suggests that IL-26 plays a role in the disease’s pathogenesis [77].

## 4. Impact of IL-20 Subfamily Members on Cancer Initiation and Progression: Pro- and Anti-Tumor Effects

IL-20 subfamily members are mainly pro-inflammatory cytokines, which can nurture an environment enabling and favoring cancer growth, playing a critical role during the multistep development of human tumors [21]. These cytokines influence tumorigenesis by regulating the hallmarks of cancer, including deregulation of proliferation, resistance to cell death, induction of angiogenesis, invasiveness, and metastasis [6].

### 4.1. IL-20 Family Members and Proliferation

Multiple lines of evidence from in vitro and in vivo experimental models suggested that IL-20 subfamily members can promote inappropriate cancer cell proliferation by regulating intracellular pathways involved in the control of cell growth and division.

#### 4.1.1. IL-19

IL-19 plays a critical role in tumorigenesis, triggering cell proliferation and mainly activating STAT3 in several cancer cells [78,79]. Upregulation of *IL-19* in an in vitro model of human BC cells induced the phosphorylation of JNK, STAT3, Erk, and AKT and an increased G2-M stage of the cell cycle, which are consistent with increased cell proliferation [49]. Similar results were reported in several other cancer models, such as skin, tongue, lung, and esophagus [38,48]. In vivo studies using an anti-IL-19 monoclonal antibody in a mouse model of SCC have demonstrated suppression of tumor growth and downregulation of *IL-19*, *TGF-β*, metalloproteinase-1 (*MMP-1*), C-X-C chemokine receptor type 4 (*CXCR4*), and Cyclin B1 expression, highlighting the therapeutic potential of targeting IL-19 in esophageal cancer [38].

#### 4.1.2. IL-20

It has been widely demonstrated that IL-20 provides an advantageous microenvironment for tumor cell growth [79]. Regarding the mechanism by which IL-20 works as a promoting factor for cellular proliferation, this cytokine stimulated the activation of JNK, STAT3, and ERK [52,80,81].

The therapeutic effects of anti-IL-20 monoclonal antibody (7E) were evaluated in oral cancer and BC in vivo models, in which 7E alleviated key malignant phenotypic characteristics by inhibiting tumor growth and tumor size [80]. More recently, the therapeutic potential of 7E has been investigated in an in vivo model of PDAC in which the antibody inhibited tumor growth and attenuated TAMs phenotype. In addition, IL-20 blockade by 7E downregulated the expression of an immunosuppressive molecule, programmed death-ligand 1 (PD-L1), which binds programmed death-1 (PD-1) on T cells, resulting in the attenuation of the activity of antitumor T cells. Finally, combined treatment with 7A and immune checkpoint inhibition with an anti-PD-1 antibody showed synergistic efficacy in decreasing tumor growth compared to either treatment alone in xenograft mice [50].

The expression of IL-20 and its receptors is subject to epigenetic regulation [32], supporting the concept that epigenetic alterations can contribute to the acquisition of hallmark capabilities during tumor development [7]. It has been shown that the induction of IL-20 expression by estrogen was epigenetically regulated in human ER-positive BC cells. Specifically, methylation of histone H3K4 at the IL-20 promoter is mediated by the selective involvement of KMT2B histone methyltransferase, determining an overexpression of IL-20. In addition, knockdown of either KMT2B or IL-20 attenuated cellular proliferation and colony formation, arresting cell cycle progression in the M-phase [82].

#### 4.1.3. IL-22

IL-22 has been associated via Stat3 activation with cancer-promoting properties in several types of cancer, including HCC, LC, and PC [83,84,85]. The effect of IL-22 on tumor growth was investigated by using an in vivo model of HCC: a significant increase in tumor volume was associated with enhanced phosphorylation of STAT3 as well as upregulation of cyclin D1 [85]. The administration of IL-22-RNAi plasmids significantly inhibited the human NSCLC cell growth in BALB/c nude mice [55]. In addition, IL-22 secreted by primary cancer-associated fibroblasts (CAFs) significantly increased the proliferation of LC cell lines, an effect partially blocked with the application of an anti-IL-22 antibody [86]. More recently, it has been demonstrated that IL-22 produced by type 3 innate lymphocytes (ILC3s) promoted proliferation of PC cells through Akt signaling activation, as IL-22/IL-22R or AKT blockage markedly counteracted such effects [87].

#### 4.1.4. IL-24

At present, IL-24 is the only cytokine of the IL-20 subfamily with tumor suppressor activity. It induces growth arrest via the JAK/STAT signaling pathway after its receptor interaction in the cell membrane, in both in vitro and in vivo models of several cancer cell lines such as melanoma, breast, lung, prostate, and pancreas [43,88,89]. The impact of IL-24 on cancer stem cells (CSCs) has been recently investigated [90]. IL-24’s ability to block proliferation and decrease the stemness of osteosarcoma CSCs was initially tested in in vitro cultured cells and was subsequently translated into an in vivo pre-clinical model by using a nude mouse xenograft. IL-24 significantly inhibited the growth of tumors originating from osteosarcoma CSCs via downregulation of both Notch and Wnt/β-Catenin signaling.

In the context of cancer gene therapy, the efficacy of recombinant mda-7 adenovirus (Ad/mda-7) was investigated in the human glioblastoma U87 cell line, in which Ad/mda-7 reduced cell proliferation and induced cell cycle arrest II [91]. Furthermore, IL-24 was integrated into chimeric antigen receptor T (CAR-T) cells (CAR.IL-24-T) to further improve the function of engineered T cells by eliminating CSCs. CAR.IL-24-T cells tested in human lung and esophageal in vitro cancer cell lines resulted in impaired cell viability and reduced sphere formation, in addition to tumor stem markers. In addition, CAR.IL-24-T cells showed superior antitumor efficacy compared to CAR-T cells in an in vivo model [92].

#### 4.1.5. IL-26

The role of IL-26 in cancer has not been studied extensively, but its involvement in the progression of human TNBC has recently been reported. IL-26 downregulation did not alter proliferation or anchorage-independent growth rates of TNBC cell lines in vitro; however, the cytokine suppressed tumor formation and growth in severe combined immunodeficiency (SCID) mice orthotopically implanted, requiring the involvement of neutrophils for this action. In addition, mice vaccination with an adenoviral agent to immunologically target IL-26 reduced tumor growth and prolonged survival, suggesting it as an actionable therapeutic target to suppress inflammation and inhibit TNBC progression [76].

#### 4.1.6. Receptor Complexes of IL-20 Subfamily Members

The role of receptors of IL-20 subfamily members in the regulation of cancer progression has been poorly studied so far. It has been reported that *IL-20RB* subunit silencing reduced the proliferation of papillary renal cell carcinoma (PRCC) [93]. Li and collaborators [94] reported that IL-20RB induced STAT3 phosphorylation to promote PC cellular stemness, as demonstrated by the increased ability to form spheroids and the high proportion of side population (SP) cells compared to the control group, while *IL-20RB* knockdown exerted opposite effects. In addition, *IL-20RB* overexpression upregulated the mRNA levels of several tumor stemness markers, including transcriptional factors such as *NANOG*, *SOX2*, and *POU5F1*, while *IL-20RB* knockdown downregulated their mRNA levels. Accordingly, similar results were obtained in a xenograft model of PC.

Analogous findings were reported for the IL-22RA1 subunit in PDAC cells, in which a sub-population of these cells, IL-22RA1^hi^, showed elevated expression of *IL-22RA1* and core stemness genes. In addition, IL-22RA1^hi^ cells displayed an elevated clonogenic activity compared to control cells both in vitro and in vivo via IL-22RA1/STAT3 signaling activation [31].

Regarding the IL-20RA subunit, it has been reported that its knockdown suppressed the cellular proliferation of a CRC in vitro model [30]. In BC cells, silencing of *IL-20RA* reduced the percentage of SP cells, an effect accompanied by decreased expression of stemness marker genes, such as *SOX2* and *OCT4*. These results were confirmed in an orthotopic allograft model in which *IL-20RA* overexpression determined an increase in tumor volume and weight compared with control mice. Mechanistically, IL-20RA promoted such effects via the JAK1–STAT3–SOX2 signaling pathway, beyond promoting a cancer-favorable immune microenvironment through the increased PD-L1 expression [13].

### 4.2. IL-20 Family Members and Resistance to Cell Death

Several studies have demonstrated that IL-20, IL-22, and IL-24 are involved in the regulation of programmed cell death by apoptosis and/or autophagy, widely implicated as physiological barriers to cancer development [12].

#### 4.2.1. IL-20

As previously reported, IL-20 was highly expressed in BC tissue and enhanced breast tumor growth in in vitro and in vivo models, triggering the activation of both signaling pathways involved in cellular proliferation (see Section 4.1.2) and antiapoptotic-associated signals such as Bcl-XL and Bad. In addition, the treatment with anti-IL-20 monoclonal antibody 7E in vivo enhanced cell apoptosis in a dose-dependent manner, further confirming IL-20’s involvement in many phases of tumor progression [80].

#### 4.2.2. IL-22

IL-22 displays a similar effect on cell death to IL-20. IL-22 contributes to human LC and PC cell survival through the upregulation of antiapoptotic proteins via STAT3 activation [55,95], an effect reversed by using antibodies against IL-22R1 subunit or transfection with the IL-22-RNAi plasmid [55]. Moreover, IL-22 reduced apoptosis via the activation of PI3K-Akt-mTOR signaling in LC cells, and the application of an anti-IL-22 antibody partially blocked this effect [86].

#### 4.2.3. IL-24

IL-24 exerts its effects not only by interacting with plasma membrane receptors but also by binding with proteins localized in the ER, mitochondria, and cytoplasm [43]. It has been reported that IL-24’s ectopic expression at supra-physiological levels induced cell death independently of the JAK/STAT pathway through several mechanisms including the unfolded protein response (UPR) related to ER stress, autophagy, apoptosis, and production of reactive oxygen species (ROS) in a wide variety of tumor cell types [43,88,89,96]. By using adenovirus vector expressing IL-24 (Ad.IL-24), it has been demonstrated that IL-24 binds ER stress markers, BiP/GRP78 and Sigma 1 Receptor (σ1), triggering mitochondrial dysfunction, ROS production, and apoptosis in human prostate cancer cell lines [97,98,99]. More recently, it has been reported that infection of human glioblastoma cell line with Ad/mda7 enhanced both the activation of apoptosis through the tumor necrosis factor-α (TNF-α) family of death receptors and autophagy, which was triggered by the upregulation of LC3-II.

#### 4.2.4. Receptor Complexes of IL-20 Subfamily Members

The involvement of IL-20RA expression in thyroid tumorigenesis was more recently reported. Silencing IL-20RA via siRNA transfection increased apoptosis by inducing p53 and Bax expression along with a decrease in Bcl-2 expression. In addition, IL-20RA expression influenced immune TME by promoting M2 macrophage polarization while reducing the polarization of M1 [100].

### 4.3. IL-20 Family Members and Angiogenesis

The ability to form new vessels is a characteristic of many tumor types. Angiogenesis is sustained by TME to obtain a supply of oxygen and nutrients in order to sustain cell viability and proliferation, as well as to allow for tumor invasion and metastasis [101].

#### 4.3.1. IL-19, IL-20, IL-22

IL-19 derived from BC cells provided a microenvironment for tumor growth stimulating the expression of MMP-2 and MMP-9, enzymes involved in tumor angiogenesis [49], while IL-22 had corresponding impact by increasing the expression of angiogenic factor, VEGF, through STAT3 activation in human PC cells, an effect reverted by using a Jak-STAT signaling inhibitor, AG490 [95]. In addition, IL-22 can act directly on endothelial cells to stimulate angiogenesis in an ex vivo mouse choroid explant model, an effect inhibited by blocking IL-22 with a neutralizing antibody [17].

Although most data suggest that IL-20 has protumor effects, it has been reported that IL-20 inhibits cyclooxygenase-2 (COX-2)/prostaglandin-E2 (PGE2) production in NSCLC by limiting COX-2-mediated angiogenesis in vitro [102]. IL-20’s anti-angiogenic properties were confirmed in a panel of NSCLC by observing a decreased expression of VEGF and its receptors [32].

#### 4.3.2. IL-24

IL-24 antiangiogenic activity was tested in several in vitro [103,104] and in vivo cancer cell models [103,104,105]. Treatment of PC cells with IL-24 reduced VEGF expression by inhibiting Src kinase activity [104], while treatment with IL-24 significantly suppressed lung tumor xenograft [103]. Accordingly, the combinatory treatment with IL-24 plus the anti-VEGF inhibitor, bevacizumab, determined reduced tumor growth in vivo [106].

### 4.4. IL-20 Family Members and Invasiveness and Metastases

TME cells evolve during tumorigenesis, acquiring an aggressive phenotype such as the ability to invade and migrate into adjacent tissue as well as into blood and lymphatic vessels in order to disseminate in several anatomical sites. Invasion and migration processes are finely orchestrated and regulated by several signaling pathways [6].

#### 4.4.1. IL-19

IL-19 enhanced BC cell migration in vitro by inducing the expression of markers involved not only in migration but also in metastasis, such as CXCR4 and fibronectin [49]. Similar results have been obtained in the human esophageal SCC cell line, in which these effects were reversed by using a monoclonal antibody against IL-19 [38].

#### 4.4.2. IL-20

IL-20 enhanced cancer cell mobility and supported invasion ability by increasing MMP-9 and MMP-12 production in breast and bladder cancer cells [52,80,81]. These effects were blocked by pretreatment with ERK1/2-specific inhibitor (U0126) or in the presence of an anti-IL-20 antibody [81].

#### 4.4.3. IL-22

IL-22 treatment promoted the invasive ability of GC cells both through STAT3 and ERK activation and upregulation of MMP-7 and MMP-13 expression, an effect abolished by the treatment with anti-IL-22 antibody [107]. Similar results were obtained in NSCLC cell lines in addition to enhanced cellular migration [83,86]. Invasion and migration were also stimulated upon the treatment of human PC cells with IL-22 secreted by ILC3s, and these effects were mediated through STAT3 and Akt signaling pathways activation [87]. By using an *IL-22* knockout BC mouse model, Katara and collaborators [108] showed that IL-22 knockout led to the arrest of the malignant transition stage through the downregulation of epithelial–mesenchymal transition (EMT)-associated transcription factors and reduced tumor growth. Additionally, IL-22 supplementation recovered BC malignancy in *IL-22* knockout mice [108].

#### 4.4.4. IL-24

IL-24 confers antitumor activity by several processes, including the inhibition of cellular migration and invasion, as observed in LC cells, an effect accompanied by the downregulation of markers such as pJNK, p38MAPK, pFAK, and MMP-2/9, associated with migration and invasion. Moreover, the authors observed a reduced number of tumor metastases in nude mice injected with LC cells, A549, transfected with the *Ad-mda7* gene [109]. IL-24 was also able to inhibit invasion, migration, and adhesion of LC cell lines in vitro by regulating the stromal-cell-derived factor (SDF)-1/CXCR-4 axis, a signaling pathway that plays a key role in LC metastasis [110].

#### 4.4.5. Receptor Complexes of Il-20 Subfamily Members

In PRCC cells, the expression of the *IL-20RB* subunit was upregulated, and its knockdown reduced the migration and invasion of cancer cells. These latter effects were accompanied by a downregulated expression of EMT hallmarks, suggesting that the IL-20RB subunit may function via the EMT to influence the invasion and migration of PRCC cells [93]. The IL-20RA subunit showed similar behavior since its knockdown reduced the migration and invasion ability of CRC cell lines [30].

**Figure 3:**
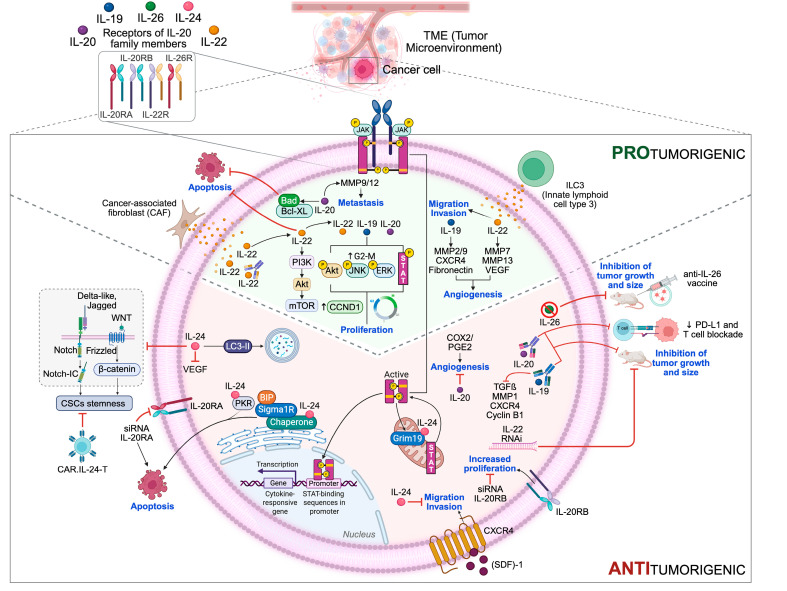
Schematic representation of data supporting the involvement of IL-20 subfamily members in the regulation of different cancer hallmarks. The main proteins and signaling pathways modulated by IL-20 subfamily members are reported. Cancer hallmarks affected by IL-20 subfamily members’ activity are shown in blue. Scheme created using BioRender.com.

Figure 3 presents a simplified overview of how the IL-20 subfamily members impact cellular events relevant to tumor development, based on the information discussed so far.

## 5. IL-20 Family/IL-20RA-Targeted Therapy: Mechanisms and Therapeutic Implications

Given that in PDAC, elevated IL-20 levels correlate with poor prognosis, preclinical studies have demonstrated that neutralization of IL-20 using monoclonal antibodies, such as 7E, inhibits tumor growth, reduces PD-L1 expression, and alleviates cachexia symptoms in mouse models. Furthermore, IL-20R1 knockdown in tumor cells also suppresses tumor progression, underscoring the receptor’s role in oncogenesis [50].

In HCC, blocking IL-20 signaling suppresses HCC cell proliferation and tumor growth in vivo, highlighting the therapeutic potential of IL-20R inhibition in liver cancer [53]. These findings suggest that targeting IL-20R may offer a novel therapeutic strategy in cancer treatment.

In this regard, ritonavir, traditionally used as an HIV protease inhibitor, has been identified as a ligand for IL-20RA and exhibits antiproliferative properties in TNBC cells. Through virtual screening of over 310,000 compounds, ritonavir was found to bind IL-20RA, and it showed anticancer activity against TNBC in vitro cell lines by significantly inhibiting cell proliferation. The study showed that preincubation with IL-20 counteracted ritonavir’s cytostatic effect. This repurposing of existing drugs underscores the potential of IL-20R1 as a therapeutic target [111].

Given the restricted expression of IL-20R in certain tissues, selective inhibition could minimize systemic immunosuppression, offering advantages over broader immunosuppressive therapies. Ongoing research is focused on developing IL-20R-specific antagonists, including monoclonal antibodies and small molecules, to evaluate their efficacy and safety profiles in clinical settings [51].

In vivo experiments utilizing the anti-IL-20 monoclonal antibody 7E have shown promising results. Treatment with 7E reduced tumor growth, suppressed bone colonization, alleviated osteolysis, and preserved bone density in murine models of BC. Additionally, IL-20R signaling has been linked to the maintenance of cancer stem cell properties through the regulation of the transcription factor SOX2, and the formation of an immunosuppressive tumor microenvironment characterized by increased expression of PD-L1 and reduced infiltration of cytotoxic T lymphocytes and dendritic cells [13]. These findings suggest that IL-20R inhibition could serve as a novel therapeutic strategy in BC, particularly in targeting metastatic and chemo-resistant disease. Ongoing research is focused on developing IL-20R-specific antagonists, including monoclonal antibodies and small molecules, to evaluate their efficacy and safety profiles in clinical settings.

Recent studies have highlighted a potential role for IL-19 in mediating hypoxia-induced CXCR4 expression in BC cells. In MCF-7 cells, exposure to the hypoxia–mimetic agent cobalt chloride (CoCl_2_) led to a marked upregulation of *CXCR4*, a chemokine receptor associated with tumor progression and metastasis. This effect was attenuated by treatment with 1BB1, a monoclonal antibody targeting human IL-19, suggesting that IL-19 contributes to the regulation of CXCR4 under hypoxic conditions. Consistently, direct exposure of MCF-7 cells to hypoxia resulted in increased *CXCR4* mRNA expression, which was significantly reduced upon treatment with 1BB1 compared to control antibodies. These findings point to IL-19 as a key mediator in the hypoxic regulation of CXCR4 and propose IL-19 inhibition as a potential therapeutic approach in BC, particularly in hypoxia-driven tumor microenvironments [112].

## 6. Conclusions

Targeting the IL-20R presents a promising therapeutic strategy in cancer therapy. Given its involvement in various aspects of tumor biology, including cell proliferation, metastasis, and immune modulation, IL-20R inhibition could complement existing therapies and improve patient outcomes. Further clinical studies are warranted to validate the efficacy and safety of IL-20R-targeted therapies in cancer treatment.
